# The Effects of K^+^ Channel Blockade on Eccentric and Isotonic Twitch and Fatiguing Contractions *in situ*

**DOI:** 10.3389/fphys.2012.00383

**Published:** 2012-09-28

**Authors:** Erik van Lunteren, Michelle Moyer

**Affiliations:** ^1^Pulmonary, Critical Care and Sleep Division, Department of Medicine, Cleveland VA Medical Center, Case Western Reserve UniversityCleveland, OH, USA

**Keywords:** 3,4-diaminopyridine, eccentric contraction, isotonic contraction, EDL, fatigue

## Abstract

K^+^ channel blockers like 3,4-diaminopyridine (DAP) can double isometric muscle force. Functional movements require more complex concentric and eccentric contractions, however the effects of K^+^ channel blockade on these types of contractions *in situ* are unknown. Extensor digitorum longus (EDL) muscles were stimulated *in situ* with and without DAP in anesthetized rats and fatigability was addressed using a series of either concentric or eccentric contractions. During isotonic protocols (5–100% load), DAP significantly shifted shortening- and maximum shortening velocity-load curves upward and to the right and increased power and work. Maximum shortening, maximum shortening velocity, and power doubled while work increased by ∼250% during isotonic contraction at 50% load. During isotonic fatigue, DAP significantly augmented maximum shortening, work, shortening velocity, and power. During constant velocity eccentric protocols (2–12 mm/s), DAP increased muscle force during eccentric contractions at 6, 8, 10, and 12 mm/s. During eccentric contraction at a constant velocity of 6 mm/s while varying the stimulation frequency, DAP significantly increased muscle force during 20, 40, and 70 Hz. The effects of DAP on muscle contractile performance during eccentric fatigue varied with level of fatigue. DAP-induced contractile increases during isotonic contractions were similar to those produced during previously studied isometric contractions, while the DAP effect during eccentric contractions was more modest. These findings are especially important in attempting to optimize functional electrical stimulation parameters for spinal cord injury patients while also preventing rapid fatigue of those muscles.

## Introduction

The fundamental strategy for successful functional neuromuscular stimulation of skeletal muscles is the use of stimulation paradigms which maximize muscle force yet minimize muscle fatigue. One common strategy has been to use variable frequency train stimulation, in which a brief high frequency burst precedes a lower frequency stimulus train, thereby eliciting the catch-like property of muscle (Binder-Macleod and Barrish, 1992; Binder-Macleod et al., 1998; Bigland-Ritchie et al., 2000; van Lunteren and Sankey, 2000). Unfortunately, this approach has not been very successful in clinical use due to only modest force increases.

Another approach is to pharmacologically increase the input-output relationship of skeletal muscle, for example by modulating membranous K^+^ channel conductances. K^+^ channels control both resting membrane potential and action potential duration, and thereby are regulators of muscle contractile performance. Of the multiple types of K^+^ channels present in muscle, voltage-gated delayed rectifier K^+^ channels play the most important roles in muscle contractile performance. Among the aminopyridines, DAP is the preferred agent due to greater potency as a K^+^ channel blocker, larger augmentation of muscle force *in vitro*, and reduced crossing of the blood-brain barrier hence smaller potential for neurologic side effects when used *in vivo* (Lundh et al., 1984; McEvoy et al., 1989; Sanders et al., 2000). In humans, DAP has been administered both orally and intravenously to treat Lambert–Eaton myasthenic syndrome, myasthenia gravis, and multiple sclerosis (Lundh et al., 1984, 1985; Sheean et al., 1988; McEvoy et al., 1989; Bever et al., 1990; Sanders et al., 2000). K^+^ channel blockers, like 3,4-diaminopyridine (DAP), augment isometric force *in vitro* and *in situ* (Miledi et al., 1977; Delbono and Kotsias, 1987; Lin-Shiau et al., 1991; van Lunteren and Moyer, 1996, 1998; van Lunteren et al., 2006, 2008; Ionno et al., 2008; van Lunteren and Pollarine, 2010). In previous *in vitro* studies, the maximum DAP-induced extensor digitorum longus (EDL) twitch force increase was 94 ± 12% (van Lunteren et al., 2006).

Due to its effects on muscle force *in vitro*, the use of the K^+^ channel blocker DAP appears to be an attractive approach to improve muscle contractile performance for *in vivo* applications and functional electrical stimulation paradigms in which high force levels are needed. However, many functional movements involve combinations of isometric, concentric, and/or eccentric contractions. An example of an upper extremity FES application is drinking. Raising the cup requires muscle shortening, holding the cup while drinking requires muscle force generation keeping muscle length constant, and lowering the cup back down requires lengthening contractions. An example of a lower extremity FES application is standing up, maintaining the standing position, and then sitting back down. Standing up requires shortening contractions, remaining standing requires isometric contractions, and sitting back down requires lengthening contractions. Walking involves predominantly shortening and isometric contractions, although lengthening contractions become more prominent during downstepping (Newham et al., 1983a,b) and downhill motion (Vihko et al., 1979; Armstrong et al., 1983a,b). Isometric, concentric, and eccentric contractions differ from each other with regards to cellular energetics and actin-myosin cross-bridge formation. It is already known that lengthening contractions result in a high incidence of contraction-induced injury, particularly more injurious than shortening contractions (McCully and Faulkner, 1985; Faulkner et al., 1989). Therefore, the likelihood of differing muscle contractile improvements to ion channel alterations through the use of a K^+^ channel blocker like DAP is quite high. Also the implications on the maintenance of any elevated force throughout a fatigue protocol are that the eccentric contractions would be less efficient. There are extensive data on aminopyridines and isometric contractions (van Lunteren and Moyer, 1996, 1998; van Lunteren et al., 2006, 2008; Ionno et al., 2008; van Lunteren and Pollarine, 2010), limited data on isotonic contractions (but only *in vitro*; van Lunteren and Pollarine, 2010), and no data on eccentric contractions.

The primary purpose of these studies is to pharmacologically increase the muscle force output during concentric and eccentric contractions using the K^+^ channel blocker, DAP. The secondary purpose of these studies is to verify that these force increases are maintained throughout a period of fatiguing stimulation. We hypothesize that the potential muscle damage due to eccentric contraction will cause DAP-induced increases in muscle contractile performance to be less than that found previously during isometric and isotonic muscle contraction. Since fatigue occurs more rapidly *in vitro* than *in situ*, we hypothesize that the muscle contractile increases *in situ* will be maintained for a longer period of time than that during the *in vitro* study. However, we also expect that DAP-induced inotropic effects during repetitive isotonic and eccentric stimulation will be shorter than that seen during isometric contraction *in situ* due to increased energy demands.

## Materials and Methods

All studies were approved by the Institutional Animal Care and Use Committee and complied with NIH animal care guidelines.

We used the fast-twitch EDL muscle preparation used previously in our lab (van Lunteren et al., 2008) and modified from Koh and Brooks (2001), in which the peroneal nerve was stimulated to induce contraction of the intact EDL muscle. A total of 39 rats (373 ± 14 g) were anesthetized with an initial 0.6 ml/kg dose of intraperitoneal ketamine (42.9 mg/ml), xylazine (8.6 mg/ml), and acepromazine (1.4 mg/ml) mixture, with supplemental doses given intraperitoneally or intravenously as needed throughout the experiment. The rats were placed on a heated blanket and maintained at a temperature of 37°C by monitoring with a rectal temperature probe. The distal tendon of the EDL was isolated and tied with silk thread. The hindlimb was stabilized by pinning the knee between sharpened screws and securing the foot. The EDL tendon was attached to the lever arm of a dual-mode servo-controlled force transducer (model 305B-L, Aurora Scientific, ON, Canada) which was controlled by a computer using a data acquisition program [Dynamic Muscle Control (DMC) Software, Aurora Scientific]. The Aurora Scientific lever system is able to operate in force-controlled or length-controlled mode so that the muscle can be stimulated in strictly isotonic, eccentric, or isometric contractions depending on the instrument settings (Watchko et al., 1997; Pollarine et al., 2007; Munkvik et al., 2008; Till et al., 2008; van Lunteren and Pollarine, 2010). The peroneal nerve was isolated and stimulated (pulse duration 0.2 ms) with a modified subminiature electrode (Harvard Apparatus, Holliston, MA, USA). The voltage used was 1.2 times that needed to achieve maximal twitch tension. Prior to the start of the protocols, using a single muscle twitch, the muscle length was adjusted to achieve maximal isometric twitch tension (*L*_0_) and kept there for the remainder of the study.

The sub-maximal tetanic force during 20–40 Hz stimulation (depending on the subsequent protocol) was determined and this value was used to calculate the percentage load when the muscle was subsequently stimulated at the respective frequency. Isometric baseline twitches were evoked at 0.1 Hz and muscle force was recorded for a period of 3 min in order to monitor muscle stability and also to provide a baseline force value for normalization. Muscles that were randomly assigned to the control or DAP treatment groups had an average baseline force of 63 ± 4 and 67 ± 3 g, respectively, and were not significantly different (*P* = 0.40). Saline (control) or DAP in saline (0.2 ml, 10 mg/kg) was infused intravenously via the jugular vein over ∼2 min, which is the same dose used in our previous *in situ* study of DAP and isometric contractions (van Lunteren et al., 2008). The optimal dose for eccentric and isotonic contractions could have been altered slightly from the optimal dose determined previously for isometric contractions. Isometric twitches were evoked at 0.1 Hz over 30 min. This incubation period was followed by either an isotonic or an eccentric protocol. Separate muscles were tested for each protocol in the absence and presence of DAP, so that drug and no drug data were obtained from muscle samples which underwent identical stimulation paradigms.

### Isotonic protocols

The subset of muscles undergoing the isotonic experiments, subsequently underwent one of two protocols. (1) *DAP Effects on Shortening-load curve* (*n* = 5, 5). Isotonic trains were evoked at 20 Hz and 1 train/min at 5–100% load (no DAP) and 5–200% load (DAP). (2) *DAP effects on Shortening During Fatigue* (*n* = 6, 8). Isotonic 40 Hz trains with a load of 40% (train duration 333 ms, 1 train/1.5 s) were evoked for a 300 cycles. The load percentages were calculated based on the maximum load in the absence of DAP.

Medium-range frequencies of 20–40 Hz were chosen because DAP has no effect on isometric force at high frequencies (Delbono and Kotsias, 1987; Lin-Shiau et al., 1991; van Lunteren and Moyer, 1996, 1998; van Lunteren et al., 2006, 2008). In preliminary experiments of fatigue during 20 Hz stimulation the amount of tetanic fusion decreased considerably during the course of repetitive stimulation, which impaired calculations of shortening velocity. We chose a stimulation frequency of 40 Hz for the isotonic fatigue experiments because this improved the duration of time over which the contractions were sufficiently fused to measure shortening velocity. The 40% load was chosen because this was the minimum load which produced the maximum amount of work, based on the results from a preliminary load curve.

### Eccentric protocols

The subset of muscles undergoing the eccentric experiments subsequently underwent one of three protocols. (1) *DAP effects on the force-velocity curve* (*n* = 7, 7). Thirty Hertz trains were evoked at 1 train/min. For the first 300 ms of each 500 ms train, the length was not changed. During the remaining 200 ms of each stimulus train, the muscle was lengthened at a velocity of 2–12 mm/s. We chose a stimulation frequency of 30 Hz based on preliminary studies indicating that this was the frequency that caused the greatest force increase during eccentric contractions. (2) *DAP effects on the force-frequency curve* (*n* = 7, 7). Trains were evoked at 1 train/min at frequencies from 1 to 70 Hz with eccentric lengthening contractions at a velocity of 6 mm/s for 0.2 s (eccentric protocol similar to that described above). (3) *DAP effects on force during fatigue* (*n* = 5, 5). Thirty Hertz eccentric trains lengthened at 6 mm/s (eccentric protocol similar to that described above) at a frequency of 1 train/1.5 s were evoked for a period of 30 min.

### Analysis

The Dynamic Muscle Analysis (DMA) High Throughput computer program (Aurora Scientific, ON, Canada) was used to measure EDL force and length changes. Muscle performance was evaluated by measuring work and power. Work was calculated as the product of the isotonic afterload and the total amount of shortening. Power was calculated as the product of the isotonic afterload (the product of the load applied to the muscle) and shortening velocity, with velocity measured during the early portion of the contraction (first 5 ms) when it was at or near its maximal value (Pollarine et al., 2007). The power data are normalized to muscle cross sectional area (muscle mass/muscle length × muscle specific gravity, assuming a muscle density of 1.06 g/ml; Close, 1972). The maximal unloaded shortening velocity (*V*_max_) was calculated based on Hill’s equation (Hill, 1938; Hatcher and Luff, 1985; Pollarine et al., 2007) by separately fitting each muscle velocity of shortening curve to Hill’s equation, determining each separate *V*_max_ and then calculating the average of all the muscles. All data were expressed as mean ± SE. Force values were normalized to the force during the pre-drug/pre-fatigue baseline period in order to correct for slight variations in rat (and EDL) size. Statistical analysis was done using repeated measures analysis of variance followed by Newman–Keuls test in the event of statistical significance. Two-tailed probability values of *P* ≤ 0.05 were considered significant.

## Results

### Isotonic contractions

**Figure 1:**
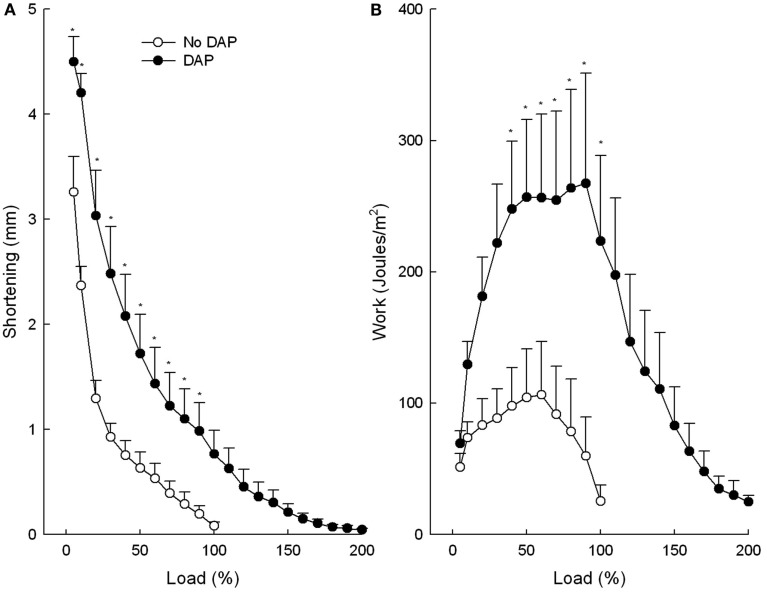
**Effects of DAP on muscle shortening (A) and work (B) during 20 Hz isotonic stimulation at different loads**. Load values were calculated as a function of the maximum load before the addition of DAP (or no drug). Values are means ± SE.

**Figure 2:**
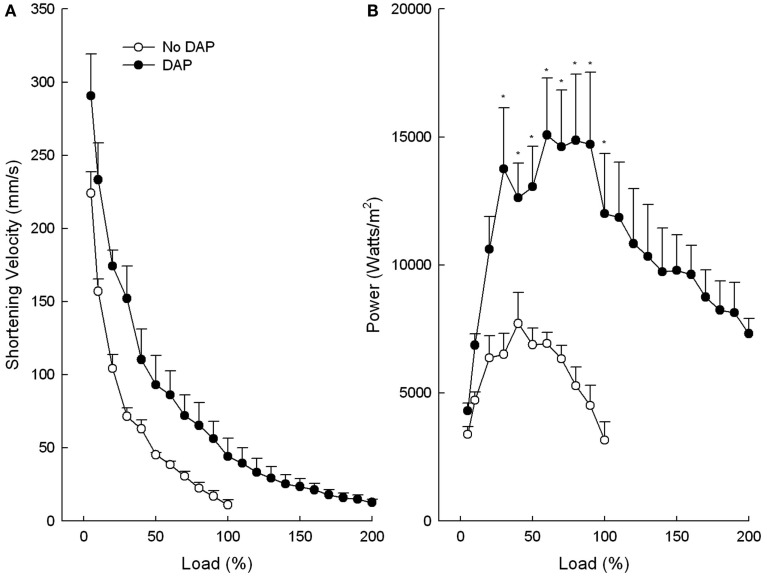
**Effects of DAP on muscle velocity of shortening (A) and power (B) during 20 Hz isotonic stimulation at different loads**. Load values were calculated as a function of the maximum load before the addition of DAP (or no drug). Values are means ± SE.

Data for shortening, work, velocity of shortening, and power were calculated as a function of the maximum load before the addition of DAP (or no drug; Figures 1 and 2). The maximum load was increased substantially by DAP which accounts for load percentages greater than 100% in the DAP-treated muscles (two-way repeated measures ANOVAs were performed only on matching data sets: load 5–100%). Values for maximum shortening and work are depicted in Figure 1. DAP shifted the relationship between shortening and load upward and to the right at all loads and was significantly increased at all loads except 100% (Figure 1A). In contrast, the effect of DAP on work varied with the load (Figure 1B). At low loads the work done by the DAP-treated muscle was nearly at the level of untreated muscle, but as the load increased the work done by the DAP muscle augmented to a significantly greater extent than that of the control muscle. Further increases in load resulted in declines in work for both groups, but the declines were right shifted for DAP-treated compared with control muscle. Finally at near-maximum loads, work was comparable for control and DAP-treated muscles.

The maximum shortening velocity curves were similar to the maximum shortening curves in that DAP shifted the curve upward and to the right at all loads, however not to the level of significant difference (*P* = 0.066; Figure 2A). The graph of power vs. load of control and DAP-treated muscles was similar in many respects to the graph of the work vs. load in that DAP did not affect power at low loads but significantly increased power at intermediate loads (Figure 2B). In contrast to work, however, power of the DAP muscles did not decrease to the level of the control muscles at very high loads. The upward shifts of the DAP relative to no drug curves were still present even when the work and power curves are plotted as a function of post-DAP load (data not shown).

The average *V*_max_ values were not significantly different between no drug and DAP (*P* = 0.33), however, the DAP values tended to be higher than control (824 ± 144 vs. 640 ± 104 mm/s, respectively). The control *V*_max_ value corresponds to 21 fiber lengths per second and this value is similar to that previously reported (20.6 fiber lengths per second) for an *in situ* EDL rat muscle (Ranatunga and Thomas, 1990).

**Figure 3:**
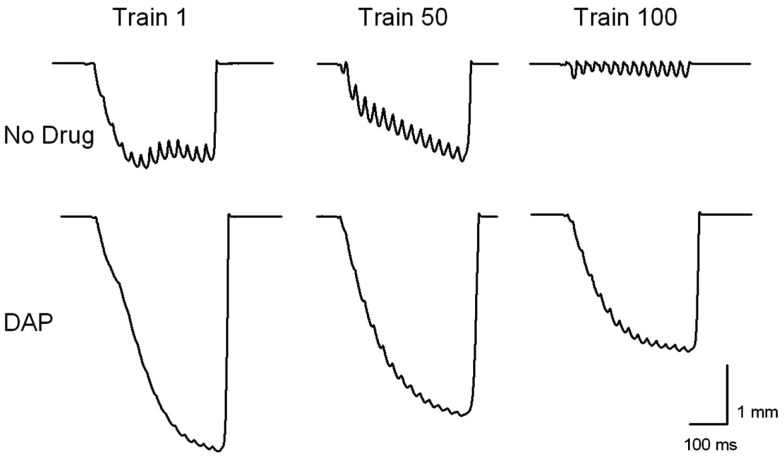
**Examples of muscle shortening during trains 1, 50, and 100 during 40 Hz intermittent isotonic contractions with and without DAP**.

**Figure 4:**
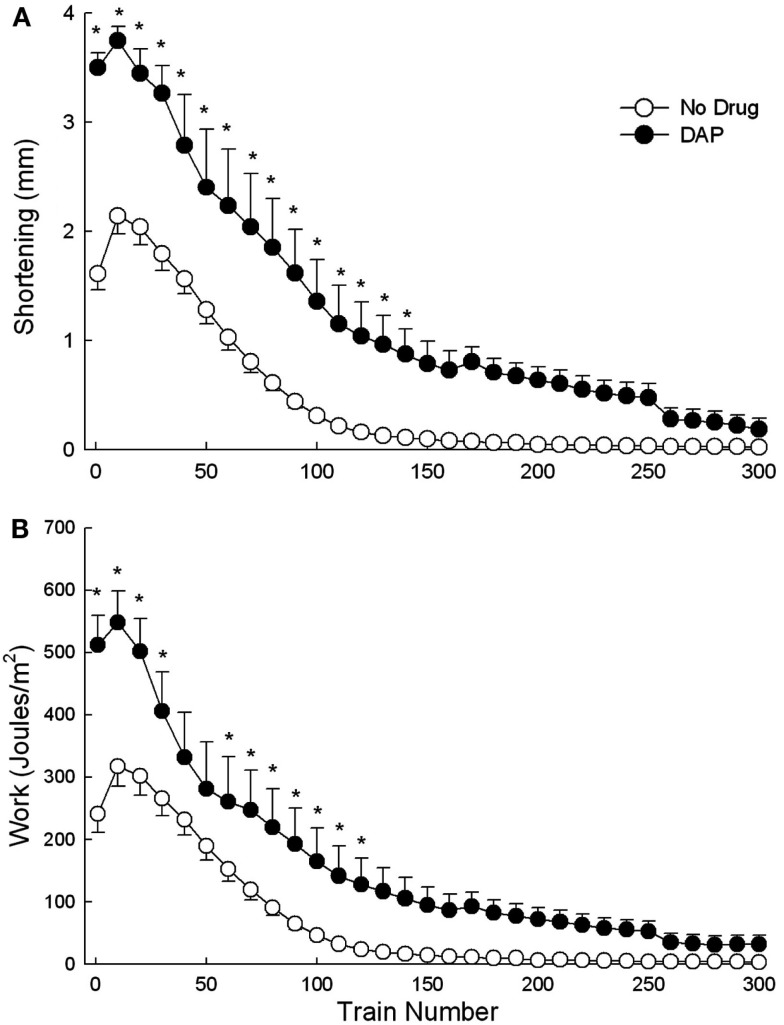
**Effects of DAP on maximum shortening (A) and work (B) during 40 Hz isotonic contraction at 40% load over the first 60 trains**. Values are means ± SE.

Figure 3 shows an example of muscle shortening during intermittent train stimulation at 40 Hz. The DAP traces indicate considerably improved contractile performance compared with control traces during the 1st, 50th, and 100th train. The maximum shortening of DAP-treated muscles was significantly greater than that of control muscles for the first 140 trains of isotonic fatiguing stimulation (Figure 4A). In addition, the work done after the addition of DAP during 40 Hz intermittent fatiguing stimulation was significantly greater than that after the addition of no drug during the 1st through 30th train and 60th through 120th train (Figure 4B). Untreated muscle had minimal shortening and work during the second half of the fatigue testing, whereas DAP-treated muscle maintained contractile performance during the same time period (Figure 4).

**Figure 5:**
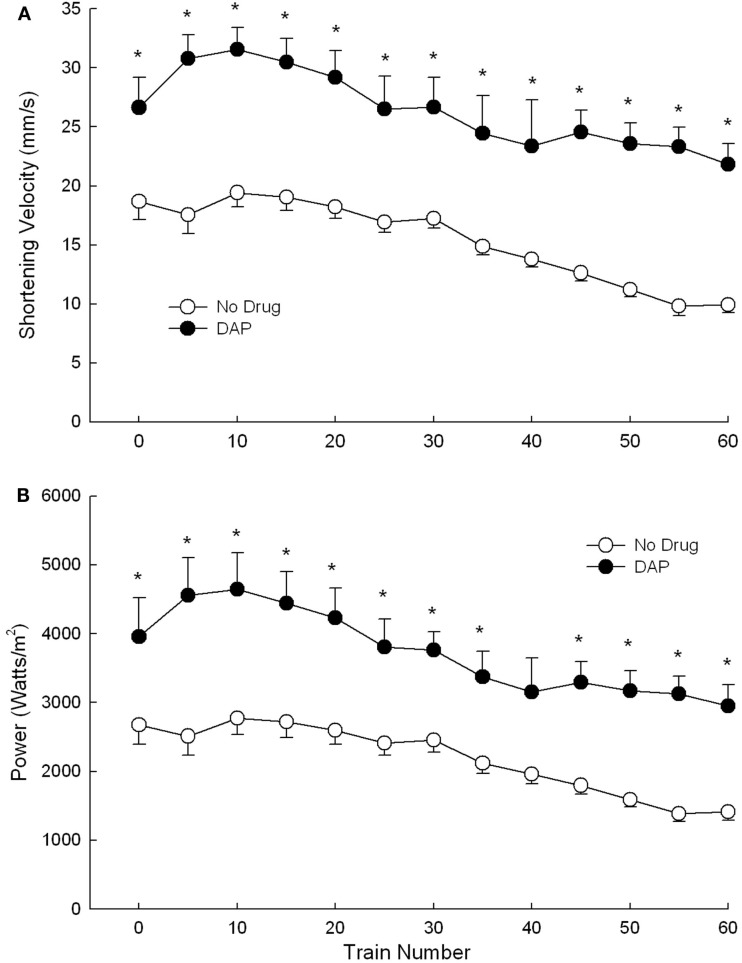
**Effects of DAP on shortening velocity (A) and power (B) during 40 Hz isotonic contraction at 40% load over the first 60 trains**. Values are means ± SE.

Shortening velocity and power were measured and calculated only during the first 60 trains of 40 Hz intermittent fatiguing stimulation because EDL muscle contraction trains became sufficiently unfused that shortening velocity (and thus also power) was not able to be measured accurately. Shortening velocity and power were both significantly greater with than without DAP throughout all (shortening velocity) or most (power) of the 60-train repetitive stimulation period (Figures 5A,B).

### Eccentric contractions

**Figure 6:**
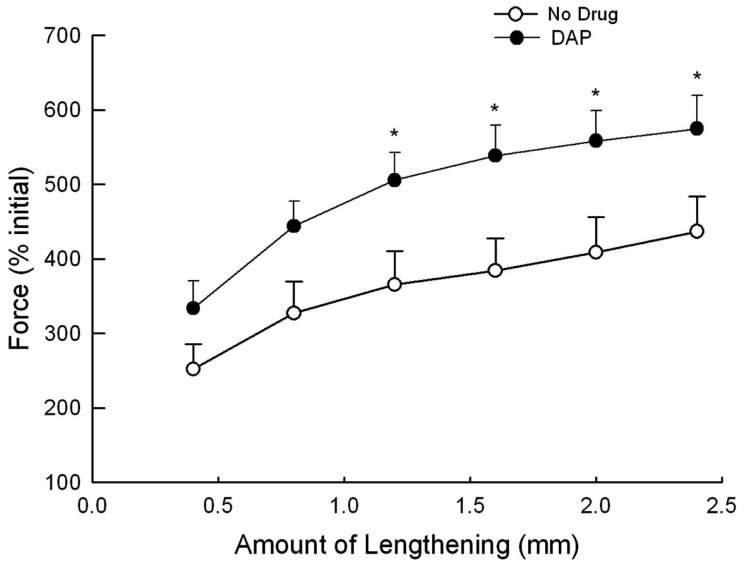
**Force during 30 Hz eccentric contraction at different lengths at a velocity of 2–12 mm/s with and without DAP**. Data are expressed as a percent of the initial pre-drug isometric twitch force. Values are means ± SE.

Eccentric contractions were assessed in separate animals than the above-described isotonic contractions. EDL muscles were lengthened by various amounts while being stimulated at 30 Hz, and the resulting muscle force during the lengthening portion of the contraction was measured to assess muscle contractile performance during eccentric contractions (Figure 6). The force produced after the addition of DAP was significantly greater than that during control conditions when muscle was lengthened by 1.2, 1.6, 2.0, and 2.4 mm when force was expressed as a percent of the initial pre-drug isometric twitch force (Figure 6).

**Figure 7:**
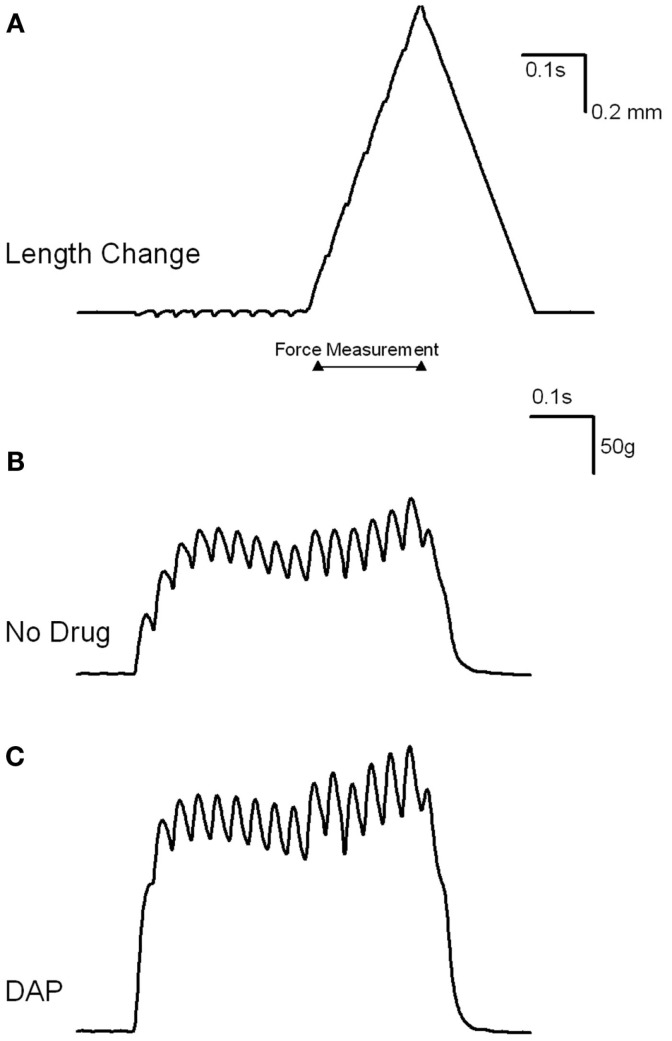
**Examples of load curve experiment. (A) Is an example of the length change from *L*_0_ to +1.2 mm**. After the first 300 ms of the 500 ms–30 Hz train, the muscle length was changed at a velocity of 6 mm/s. Peak force was measured while the muscle was being lengthened and stimulated (indicated by flat line). **(B)** Shows the resulting change in load after the addition of control saline. **(C)** Shows the resulting change in load after the addition of DAP.

**Figure 8:**
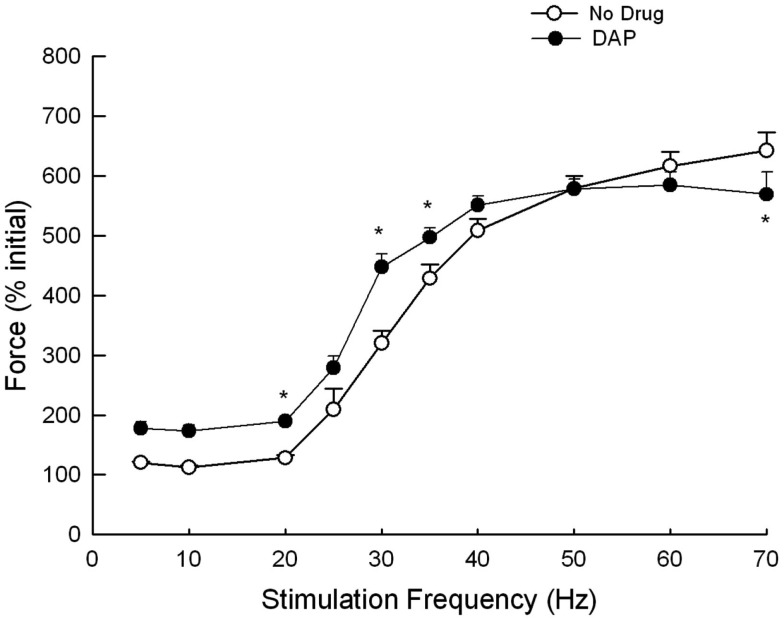
**Force during eccentric contractions of 1.2 mm at constant velocity of 6 mm/s at different frequencies with and without DAP**. Data are expressed as a percent of the initial pre-drug isometric twitch force. Values are means ± SE.

Muscles were also stimulated at various frequencies while the amount of lengthening was constant (1.2 mm), and the resulting muscle force is depicted in Figures 7 and 8. Figure 7 is an example of the eccentric contractions. After the first 300 ms of the 500 ms–30 Hz train, the muscle length was changed at a velocity of 6 mm/s. Muscle force was significantly greater with the addition of DAP at frequencies of 20, 30, and 40 Hz when calculated as a percent of initial force (Figure 8).

**Figure 9:**
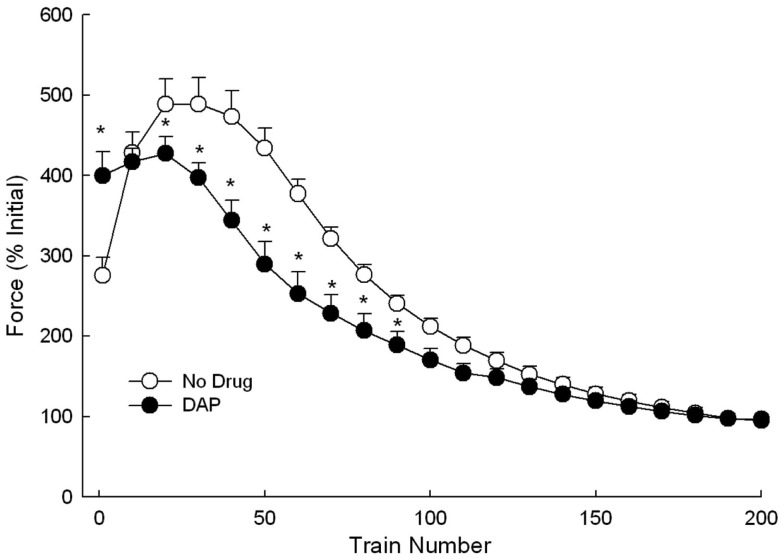
**Force during repeated 1.2 mm 30 Hz eccentric contractions at constant velocity of 6 mm/s with and without DAP**. Values are expressed as a percent of the initial pre-drug isometric twitch force. Values are means ± SE.

Eccentric fatigue testing was done during stimulation at 30 Hz while lengthening the muscle by 1.2 mm (Figure 9). The initial 30 Hz muscle force was significantly greater with DAP than without. Second, the control muscles exhibited a larger amount of force potentiation than the DAP muscles. After the 10th cycle, the potentiation resulted in a significantly higher control muscle force than the DAP muscle force (Figure 9). Third, the control muscle force remained significantly higher than that of DAP muscle force until the 100th cycle, when it had decreased to levels that were no longer significantly different than that of DAP muscle force.

## Discussion

During isotonic contractions, maximum shortening, maximum shortening velocity, and power doubled while work increased by ∼250%. These parameters remained augmented throughout the period of 40 Hz isotonic fatigue. During eccentric contraction, DAP significantly increased muscle force during 20, 40, and 70 Hz. However, the effects of DAP on muscle contractile performance during eccentric fatigue varied with level of fatigue. DAP-induced contractile increases during isotonic contractions were similar to those produced during previously studied isometric contractions, while the DAP effect during eccentric contractions was more modest.

Many types of K^+^ channels are present in mammalian skeletal muscle which control both resting membrane potential and action potential duration, and thereby are key regulators of muscle contractile performance. The findings of this study are especially important in attempting to optimize functional electrical stimulation parameters for spinal cord injury patients while also preventing rapid fatigue of those muscles.

The present study utilized intermediate stimulation frequencies for this study because augmentation of isotonic contractile performance with DAP was documented at low to intermediate stimulation frequencies in previous *in vitro* studies (Miledi et al., 1977; Delbono and Kotsias, 1987; Lin-Shiau et al., 1991; van Lunteren and Moyer, 1996, 1998; van Lunteren et al., 2006, 2008; Ionno et al., 2008; van Lunteren and Pollarine, 2010). In addition, we have consistently found that DAP and the closely related 4-aminopyridine do not improve isometric force at high stimulation frequencies either *in situ* or *in vitro* (van Lunteren et al., 1995, 2006, 2008; van Lunteren and Moyer, 1996, 1998; van Lunteren, 1999; Ionno et al., 2008; van Lunteren and Pollarine, 2010). Most importantly, almost all functional neuromuscular stimulation paradigms use low to intermediate rather than high stimulation frequencies for restorative tasks.

The lack of force increase produced by the aminopyridines at high stimulation frequencies potentially limits their clinical utility for muscle weakness due to primary muscle disease. However, during functional neuromuscular stimulation applications designed to restore motor activity in subjects with spinal cord injuries, modest rather than high stimulation frequencies are frequently used (Glenn et al., 1988; Bhadra and Peckham, 1997). Some restorative applications are currently limited by the need to generate high force values while at the same time avoiding muscle fatigue, in particular for weight-bearing activities such as standing up from a seated position, maintaining a standing posture, and walking. Several electrical stimulation paradigms have been devised to optimize the input-output relationship of skeletal muscle, such as variable frequency stimulation (Burke et al., 1970, 1976; Binder-Macleod and Barrish, 1992; Bigland-Ritchie et al., 2000), but this has had limited clinical effectiveness in human functional neuromuscular stimulation applications due to relatively modest force increases, in particular when compared with the force augmentation that can be achieved with pharmacological agents like DAP.

These complex functional tasks often involve shortening contractions and lengthening contractions in addition to static contractions, often with different muscles performing different types of contractions, but also with some muscles performing different types of contractions during different phases of the task (Dickinson et al., 2000; Rome, 2002). There are many studies that demonstrate the increased energy demands and excess heat production of isotonic relative to isometric contractions (Hill, 1938; Han et al., 2003) and the damaging effect that repetitive and excessive eccentric contractions have on muscle fibers (Asp et al., 1995; Fluckey et al., 2001). Thus, it is important to augment muscle force for the shortest amount of time possible to prevent the least amount of muscle fiber damage from excessive stimulation.

In the present study, maximum shortening, work, shortening velocity, and power were all increased due to DAP during isotonic contractions. The amount of increase is difficult to compare directly to the results from previous experiments due to the different parameters used to measure the different types of contractions, but nonetheless comparisons of percentage increases in contractile performance, as well as durations of contractile improvements during repetitive stimulation, produced by DAP can be informative.

In previous studies, twitch force increases of ∼75–150% were seen during *in vitro* isometric contraction at optimal DAP concentrations in normal muscle (van Lunteren and Moyer, 1996, 1998, 2002). The duration of DAP force augmentation during isometric *in vitro* studies was typically in the order of 2–5 min (van Lunteren and Moyer, 1996, 1998, 2002). *In situ* isometric contraction force percent increases were similar in magnitude to the isometric *in vitro* increases (van Lunteren et al., 2008). Twitch force increased by 105% and the initial train force during 20 Hz fatigue was elevated by 110%, staying elevated by 25% throughout the remaining 30 min of intermittent *in situ* isometric contraction stimulation. In our recent *in vitro* isotonic contraction experiments DAP improved both work and power of the diaphragm and fast-twitch leg muscle (van Lunteren and Pollarine, 2010). These increases occurred throughout the various loads tested, but were greatest at 60% load. For the EDL, work increased by ∼200% and power increased by ∼500%.

In the current study, DAP increased *in situ* isotonic contractions similar to the previous studies as evident by a 200% maximum increase in power at 90% load and a 600% increase in work at 100% load. At the onset of the fatigue run, DAP increased maximum shortening by 150%, work by 115% and shortening velocity, and power by 40% each. These increases became smaller throughout the 300 train stimulation period and approached the levels of untreated muscles by the end of the 7.5 min protocol.

DAP increased muscle force during lengthening contractions by a maximum of 85% during the lengthening curve. There have been no eccentric EDL muscle contraction studies done *in vitro* or *in situ* with DAP to compare to the present study. However, this is comparable to the large force increases seen in isometric *in situ* contractions. During fatigue, the initial force was ∼100% higher with DAP, however this force quickly decreased and fell below that of untreated muscles after less than a minute of stimulation, in contrast to both isometric and isotonic fatiguing contractions during which contractile performance of DAP-treated muscle never declined below that of untreated muscle (van Lunteren et al., 2008; van Lunteren and Pollarine, 2010).

Blood pressure was not monitored during this experiment, and it is possible that blood pressure or more importantly blood flow are altered by DAP in the dose we used. Alterations in blood pressure or blood flow could contribute to the beneficial effects of DAP. However, DAP was also found previously to increase muscle performance *in vitro* (van Lunteren and Moyer, 1996, 1998; van Lunteren et al., 2006, 2008; Ionno et al., 2008; van Lunteren and Pollarine, 2010), indicating that DAP has substantial direct effects independent of blood flow.

In conclusion, DAP augments both isotonic and eccentric EDL muscle contraction *in situ* similar to previous *in vitro* studies. However, these increases were not as long-lasting as the increases seen during *in situ* isometric contractions during the course of fatigue-inducing contractions. Perhaps the extra energy required for isotonic contractions and the muscle damage that occurs during eccentric contraction prevent these increases from lasting as robustly throughout the course of stimulation as was seen previously with isometric contractions (van Lunteren and Moyer, 1996, 1998, 2002). The difference in the amount of eccentric contraction muscle damage between DAP-treated and control muscle was not measured. The effects of DAP on the amount of protection to this injury is a subject worthy of future experimentation. The benefits of DAP treatment are greater during isometric and isotonic contractions, however there is still force augmentation during eccentric contractions. The benefit during eccentric contractions is early and vanishes as the muscle becomes fatigued. It is clear from these results that DAP-induced augmentation of contractile force is still effective during isotonic and eccentric contractions and will have potential therapeutic value in functional electrical stimulation applications.

## Conflict of Interest Statement

The authors declare that the research was conducted in the absence of any commercial or financial relationships that could be construed as a potential conflict of interest.

## Appendix

**Table A1:** **Force (in grams) during 30 Hz eccentric contraction at different lengths at a velocity of 2–12 mm/s, during eccentric contractions of 1.2 mm at constant velocity of 6 mm/s at different frequencies and during repeated 1.2 mm 30 Hz eccentric contractions at constant velocity of 6 mm/s with and without DAP**.

	No drug	DAP
**Lengthening (mm)**
2	150 ± 23	194 ± 19
4	193 ± 27	260 ± 20
6	214 ± 27	295 ± 19
8	227 ± 28	314 ± 20
10	239 ± 28	326 ± 20
12	256 ± 27	335 ± 21
**Frequency (Hz)**
5	78 ± 7	113 ± 8
10	73 ± 6	110 ± 8
20	83 ± 8	121 ± 9
25	141 ± 35	182 ± 24
30	208 ± 23	286 ± 23
35	270 ± 27	320 ± 24
40	318 ± 22	352 ± 21
50	365 ± 22	369 ± 22
60	390 ± 22	373 ± 23
70	411 ± 26	362 ± 30
**Fatigue (s)**
1	166 ± 19	274 ± 18
10	264 ± 37	285 ± 6
20	297 ± 38	292 ± 8
30	296 ± 36	271 ± 5
40	285 ± 32	234 ± 8
50	261 ± 25	196 ± 12
60	227 ± 20	171 ± 12
70	193 ± 16	155 ± 11
80	167 ± 15	141 ± 10
90	145 ± 14	128 ± 8
100	129 ± 14	116 ± 7
110	115 ± 14	105 ± 5
120	103 ± 13	102 ± 8
130	93 ± 12	94 ± 7
140	86 ± 11	88 ± 6
150	78 ± 10	82 ± 4
160	73 ± 9	77 ± 4
170	68 ± 8	73 ± 3
180	64 ± 7	69 ± 3
190	59 ± 7	67 ± 2
200	58 ± 7	66 ± 3
